# Effect of Low Amperage Electric Current on Staphylococcus Aureus—Strategy for Combating Bacterial Biofilms Formation on Dental Implants in Cystic Fibrosis Patients, In Vitro Study

**DOI:** 10.3390/ma14206117

**Published:** 2021-10-15

**Authors:** Anna Minkiewicz-Zochniak, Kamila Strom, Sylwia Jarzynka, Bartłomiej Iwańczyk, Anna Koryszewska-Bagińska, Gabriela Olędzka

**Affiliations:** 1 Department of Medical Biology, Medical University of Warsaw, Litewska 14/16, 00-575 Warsaw, Poland; anna.minkiewicz@wum.edu.pl (A.M.-Z.); kamila.strom@wum.edu.pl (K.S.); sylwia.jarzynka@wum.edu.pl (S.J.); anna.koryszewska-baginska@wum.edu.pl (A.K.-B.); 2 Department of Oral Surgery, Medical University of Lublin, Karmelicka 7, 20-081 Lublin, Poland; dent.iwanczyk@gmail.com

**Keywords:** cystic fibrosis, *Staphylococcus aureus*, electric current, dental implants, titanium and zirconium

## Abstract

Cystic fibrosis is an inherited disease that affects multiple organs and systems. The oral cavity can serve as a substantial source of bacteria, causing respiratory infections and diseases which continue to dictate the clinical course of the disease and prognosis in patients with CF. Low voltage and electric current could effectively kill bacteria and biofilms, and the activity of milliampere currents could be used as an effective method of fighting bacteria. This study evaluated the effect of low amperage electric current on the formation of *Staphylococcus aureus* biofilms on dental implants such as titanium and zirconium in patients with cystic fibrosis. Our studies suggest that a constant electric current at a low intensity of 1 mA and 10 mA is inhibiting bacterial adhesion, detaching biofilm-forming bacteria on biomaterials used in dental implants such as titanium and zirconium, and destroying bacterial cells of *Staphylococcus aureus* strains. In addition, we observed the selection of an appropriate biomaterial for implants in people affected by chronic diseases, such as CF, should be carefully planned.

## 1. Introduction

Cystic fibrosis (CF) is an inherited recessive disease caused by multiple mutations in a single cystic fibrosis transmembrane conductance regulator (CFTR) gene [1]. The evidence of this common disorder in the European Union has long been estimated at 1 in 2000–3000 newborns [2]. CF displays different clinical expressions. However, changes in the pulmonary and digestive system are predominant [3]. Common lung infections often lead to severe and life-threatening forms and are responsible for the majority of death in CF patients [3,4,5,6]. Although CF remains incurable, advancements in medicine and the implementation of multidisciplinary care have extended median survival from less than 10 to over 45 years in developed countries [1,7].

The human oral cavity microbiome consists of a number of different groups of microorganisms, either as planktonic cells or incorporated into diverse biofilms [8,9]. Numerous studies have revealed significant differences in both the number and the overall composition of oral microorganisms of CF and non-CF individuals. It is also the main reservoir of potentially pathogenic bacteria, including *S. aureus*, *P. aeruginosa*, and some *Enterobacteriaceae* [10,11]. In the most recent publication on this subject, bacterial genera such as *Chryseobacterium*, *Microbacterium*, *Brevundimonas*, *Stenotrophomonas*, *Streptococcus, Rothia*, *Staphylococcus*, *Delftia*, *Comamonas*, *Scardovia*, *Mobiluncus Sphingobacterium*, *Mogibacterium*, and the fungal species *Candida albicans* were identified at higher abundance and prevalence in the oral rinse samples of CF patients [12]. Conversely, smaller amounts of the genera *Treponema*, *Peptostreptococcus*, *Alloprevotella*, *Aggregatibacter*, *Parvimonas*, *Bergeyella*, *Fusobacterium*, genera of the order *Saccharimonadales*, and *Clostridiales* family were found [10,11,12]. Impaired homeostasis in the oral cavity of CF patients may also be associated with a reduced amount and concentration of saliva [13]. The degree of microbial colonization of the oral cavity may result in the development of stomatitis, gingivitis and periodontitis, and dental plaque and the formation of tooth and implant biofilms that initiate periodontal disease and caries. There have been various reports of dental conditions in individuals with CF, most of which relate to pediatric patients [14]. Despite the strong tendency for dental calculus formation, individuals with CF are less likely to get gingivitis [15]. It can be also hypothesized that due to the high levels of *Streptococcus mutans*, this group is at higher risk of dental caries [16]. Additionally, the increased energy demand in patients with CF necessitates therapy and nutritional support treatment that promotes the development of caries and increases the severity thereof. However, some studies suggest that, despite creating a low PH and low alpha diversity environment favoring damage to the outer layers of the teeth, the combination of CF common factors can also inhibit cariogenic and periodontal pathogens [12].

A number of oral bacterial taxa can affect not only oral health but also contribute to the initiation and/or progression of certain types of inflammation in the respiratory tract of CF patients [11,17,18]. Poor oral health may affect lung function and increase the risk of bacterial pneumonia [17]. Although *P. aeruginosa* and *B. cepacia* bacterial complexes are the most common pathogens associated with a shorter life of CF patients, they do not colonize the airways alone. One such microorganism is methicillin-resistant *S. aureus* (MRSA) [19,20], whose infections are more prevalent in young adults who have undergone many years of antibiotic therapy. Patients with CF and confirmed MRSA have a higher rate of hospitalization, and a one-third greater risk of death compared to MRSA-negative patients [19,21]. It is also assumed that continuous prophylactic and conservative use of oral, inhaled, and intravenous antibiotics against *Staphylococcus* spp., may increase the risk of early irreversible colonization by *P. aeruginosa*, the main pathogen of patients with CF [22].

Bacterial respiratory infections continue to dictate the clinical course of the disease and prognosis in patients with CF. Their targeted and aggressive treatment with antibiotics is widely believed to be the main reason for the increase in life expectancy in CF patients [22]. However, in cases of end-stage lung disease transplantation of this organ remains the only therapeutic option [23,24]. It is recommended that before this method of treatment candidates should be declared “dentally fit” and therefore often require dental treatment [25]. These recommendations particularly apply to CF patients who have decided to use dental implants due to missing teeth and are at increased risk of peri-implantitis. An unhygienic oral environment and resulting oral disease can negatively impact systemic health [26,27], and contribute to the reduction of the survival rate due to the occurrence of infectious complications, which account for more than 37% of reported deaths in the first year after lung transplantation and about 22% of deaths after the third year [23].

According to our knowledge, there is no clear evidence-based guidelines on the dental management of adult patients with CF. Therefore, our study aimed to evaluate the effect of a non-invasive method of using low-intensity direct current on biofilms formed by *S. aureus* collected from the sputum samples of adult CF patients. Similar methods, albeit based on the germicidal effects of high voltage and current, are already known in dentistry [28]. Nevertheless, it has also been confirmed that low voltage and electric current, and even ultra-low electric current at microamperes and/or milliamps, could effectively kill bacteria and biofilms. Therefore, they can be used to effectively reduce bacterial biofilms [29,30,31,32,33]. However, the effectiveness of the low voltage currents in treating dental plaques is unclear. For this reason, in our research, we focused on the selection of the most bactericidal time and intensity of low amperage electric current so that patients, as part of prophylaxis, could effectively prevent infection in the dentist’s office. Furthermore, we studied how the antibiotic therapy of CF patients in the case of inflammation around the implant may affect the clinical course of the disease and its long-term prognosis.

## 2. Materials and Methods

### 2.1. Bacterial Strain Selection and Storage Conditions

Among the 33 clinical strains of *S. aureus* identified in previous studies [34], 3 clinical strains of *S. aureus* were selected with their ability to form a biofilm classified as strong, moderate, weak, and non-producers. These strains were obtained from the sputum samples of patients diagnosed with CF and treated at the Department of Microbiology, National Tuberculosis and Lung Diseases Research Institute (Warsaw, Poland), Culture Collection, Department of Medical Biology, Medical University of Warsaw, Poland). All strains were stored frozen at −70 °C in lysogeny broth (LB) medium (Sigma-Aldrich, St Louis, MO, USA) with 2% glycerol prior to the study.

### 2.2. Biofilm Assay and Strain Classification

The strains of *S. aureus* were assessed for their ability to form biofilms and were classified as strong, moderate, weak, and non-producers. The colorimetric assay of biofilm formation using crystal violet staining was performed as previously described [34,35,36].

### 2.3. Preparation of Biomaterial Discs

Biomaterial discs were prepared in accordance with the standard procedures of the dental laboratory and the manufacturer’s recommendations. We made round discs with a diameter of 10 mm and a thickness of 2 mm from titanium alloy Ti-6Al-4V (grade 5 titanium) and zirconium dioxide (yttria-stabilized tetragonal zirconia polycrystals), which are commonly used in clinical practice. Original materials were obtained from Silesia Dental (Środa Śląska, Poland).

Each disc was cleaned in an ultrasonic bath (POLSONIC, Warsaw, Poland) in a solution containing 1% Extran^®^AP 15 (Merck KGaA, Darmstadt, Germany) and 99% deionized ultrapure water (HYDROLAB, Straszyn, Poland) for 15 min. The ultrapure water was then rinsed three times and ultrasonicated for 15 min, followed by rinsing in 99.8% ethyl alcohol (Avantor, Gliwice, Poland) for 15 min. Before biofilm formation assay, the discs were treated with dry heat sterilization.

### 2.4. Biofilm Eradication Assay

Sterile Ti-6Al-4V discs and zirconium dioxide were inserted into 500 mL of LB medium supplemented with 0.2% glucose in a conical flask. Subsequently, they were inoculated with *S. aureus* to a final concentration of 10^5^CFU/mL^−1^. The concentration of bacteria was determined using a spectrophotometer with an automated Synergy HTX multi-mode reader (BioTek Instruments Inc., Winooski, VT, USA), and growth was allowed to proceed for 24 h at 37 °C in a shaker at 160 rpm. The discs were aseptically removed from the cultures, rinsed in three baths of 10 mL saline buffer to remove non-adherent cells, and then transferred to 500 mL phosphate-buffered saline (PBS) buffer. The discs were then pretreated using low amperage electric current electric stimulation for 0, 10, and 60 min. Immediately after that, discs were aseptically removed from the cultures, biofilms were washed twice with 10 mL saline buffer to remove non-adherent cells, and vortexed. Subsequently, the total number of bacteria in each sample was determined using the viable plate counting method. The experiment was performed in duplicate and repeated three times independently.

### 2.5. Biofilm Inhibition Assay

To assess biofilm inhibition, Ti-6Al-4V discs and zirconium dioxide were immersed in a conical flask containing 500 mL of *S. aureus* suspension, respectively, in LB medium supplemented with 0.25% glucose at 37 °C for 2 h to induce preliminary adhesion of cells to the surface. The discs were then pretreated with electric stimulation for 0, 10, and 60 min. Immediately after that, discs were aseptically removed from the cultures, rinsed twice in 10 mL saline buffer to remove non-adherent cells, and vortexed. Subsequently, the total number of bacteria in each sample was determined using the viable plate counting method. The experiment was performed in duplicate and repeated three times independently.

### 2.6. Electrical Stimulation

Low amperage electric current electrical stimulation was provided at a current of 1 mA and 10 mA using a power supply (Consort EV 243, Sigma, Poland) connected to the electrodes emerging from the chamber. Electrical stimulation was applied for 0, 10, and 60 min. The stimulation output was controlled by the current.

### 2.7. Biofilm Detachment from Surfaces and Assessment of Bacterial Viability

The bacterial cells attached to the surface of the biomaterial discs were counted after biofilm formation colony-forming unit (CFU) counting assay was used. The discs were placed in 15 mL Falcon tubes (Falcon^®^ Conical Tubes, STEMCELL, Renosa, Mexico) containing 10 mL PBS buffer and vortexed twice at 30 × g for 3 min to dislodge the adherent bacteria. Tenfold serial dilutions of up to 10^−6^ were prepared in PBS buffer. Spread plating (double tests) was performed on Chapman agar plates using 100 µL of the undiluted and six dilution samples. CFU counting was performed after incubation at 37 °C for 24 h.

### 2.8. Visualization of Biofilm Eradication Assay with Low Amperage Electric Current Electrical Stimulation in Fluorescence Microscopy

Sterile glass coupons were inserted into 500 mL of LB medium supplemented with 0.2% glucose in a conical flask. Then, they were inoculated with S. aureus to a final concentration of 10^5^ CFU/mL^−1^, and growth was allowed to proceed for 24 h at 37 °C in a shaker at 160 rpm. The glass coupons were aseptically removed from the cultures, rinsed in three baths of 10 mL saline buffer to remove non-adherent cells, and then transferred to 500 mL PBS buffer. The glass coupons were then pre-treated using low amperage electric current electric stimulation for 0, 10, and 60 min. Immediately after that, glass coupons were aseptically removed from the cultures, biofilms were washed twice with 10 mL saline buffer to remove non-adherent cells were examined for bacterial viability using fluorescence microscopy. The specimens were stained with the LIVE/DEAD kit according to the manufacturer’s instructions (LIVE/DEAD™ BacLight™ Bacterial Viability Kit, Molecular Probes, Art. No. L7012, Invitrogen, Molecular probes, Eugene, OR, USA) and examined using fluorescence microscopy (OPTA-TECH, Warsaw, Poland). Each experiment was performed under identical conditions at different times. Each of glass discs without control (after 10 min, 60 min) were pretreated with electric stimulation in experiments with 1 mA and experiments with 10mA. The LIVE or DEAD images were taken for each sample separately.

### 2.9. Statistical Analyses

The results are presented as mean ± standard deviation. All data were statistically analyzed from three independent experiments using the Student’s *t*-test. Statistical significance was set at *p* < 0.05. Statistical analyses were performed using Statistica 13.3 (version 13.3,TIBCO Software Inc., Palo Alto, CA, USA), licensed to the Medical University of Warsaw.

## 3. Results

### 3.1. Effect of Low Amperage Electric Current of 10 mA on Biofilm Formation on Ti-6Al-4V and Zirconium Dioxide Discs

The strains showed differential abilities to form biofilms on dental biomaterials after 24 h of incubation, consistent with a previous study. Based on our observations, the strains were categorized as weak biofilm (WB), moderate biofilm (MB), or strong biofilm (SB) producers, as previously described and standardized.

**Figure 1:**
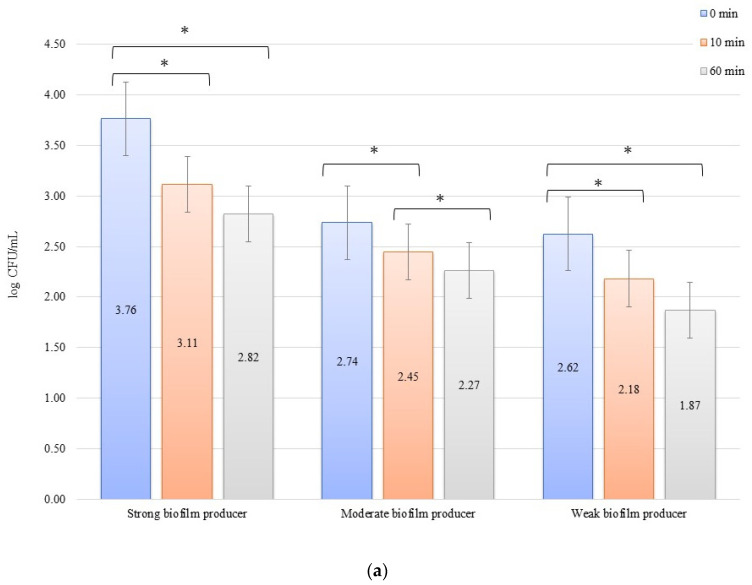

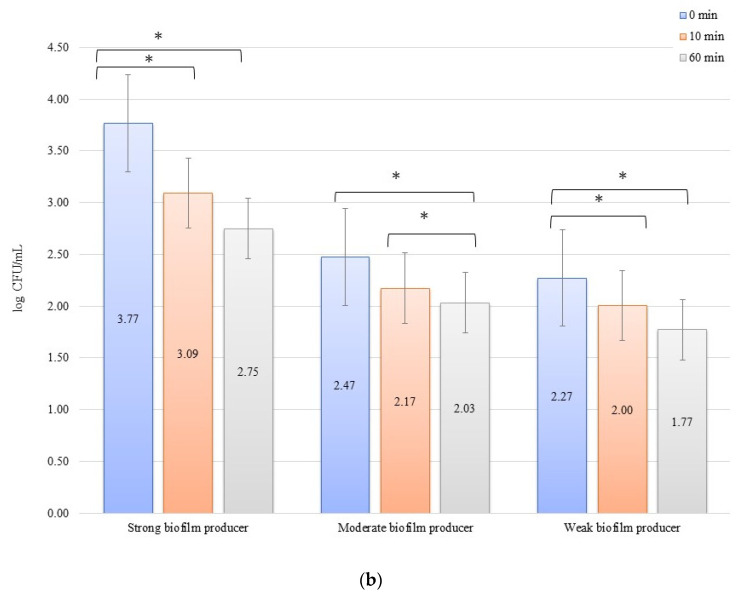
Biofilm eradication assay for *Staphylococcus aureus*. Strains forming biofilms on Ti-6Al-4V (**a**) and zirconium dioxide (**b**) discs classified as strong, moderate, and weak are treated with low direct electric current of 10 mA. Error bars represent the pooled standard deviations of the mean. The level of significance is presented at * *p* < 0.05. The mean and standard deviations are shown.

The numbers of bacterial cells forming biofilm after 24 h on the zirconium discs in the erosion experiment were 3.77, 2.47, and 2.27 log CFU/mL for SB, MB, and WB in 0 min, respectively. On the titanium disc, these were 3.76, 2.74, and 2.62 log CFU/mL for SB, MB, and WB, respectively. A decrease in the number of bacteria was observed over time after exposure to an electric current of 10 mA (Figure 1a,b). In all cases of SBs, MBs, and WBs, taking into account the exposure time of the biofilm formed on both the zirconium and titanium discs in low amperage electric current electrical stimulation erosion, the reduction of bacterial cells in the biofilm was significant. The decrease in the number of cells was statistically significant (*p* < 0.05) after 10 min of erosion on the zirconium disc (time 0 min to 10 min) for SB (*p* = 0.0048) and WB (*p* = 0.002). After another 50 min of erosion (time 10 min to 60 min), a statistically significant reduction in the number of bacterial cells was observed only in MB (*p* = 0.0129). Within 0 min to 60 min, the results for each biofilm were statistically significant: SB, *p* = 0.016; MB, *p* = 0.039; and WB, *p* = 0.026. Erosion on the titanium discs showed a statistically significant decrease in the number of cells (*p* < 0.05) after 10 min (time 0 to 10 min) for SB (*p* = 0.009), MB (*p* = 0.030), and WB (*p* = 0.0129), and after 50 min (10 to 60 min), the reduction in MB was statistically significant (*p* = 0.021). The dependence of biofilm reduction from 0 to 60 min was statistically significant for SB (*p* = 0.0039) and WB (*p* = 0.0018).

**Figure 2:**
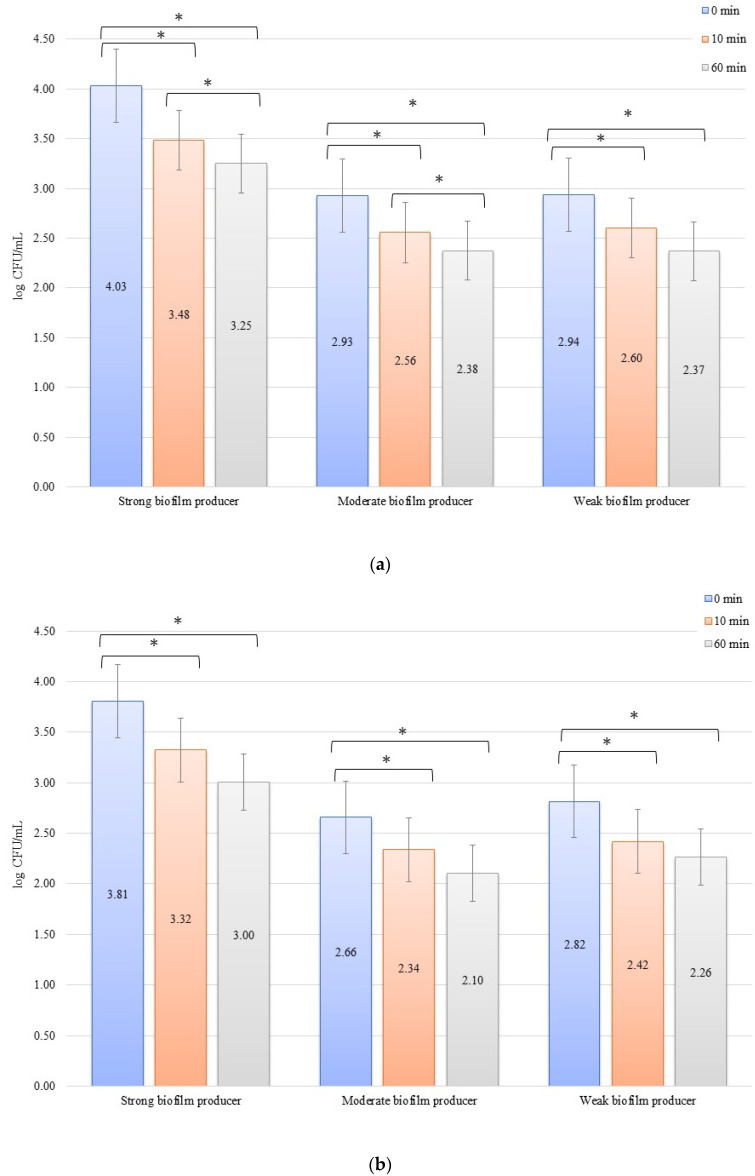
Biofilm inhibition assay for *Staphylococcus aureus*. Strains forming biofilms on Ti-6Al-4V (**a**) and zirconium dioxide (**b**) discs classified as strong, moderate, weak, and non-producers are treated using low direct electric currents 10 mA. Error bars represent the pooled standard deviations of the mean. The level of significance is presented at * *p* < 0.05. The mean and standard deviations are shown.

For the inhibition, the initial numbers of biofilm cells (Figure 2a) on the zirconium discs were 3.81, 2.66, and 2.82 log CFU/mL for SB, MB, and WB, respectively. The growth of bacterial biofilm was noted on the titanium discs (Figure 2b): 4.03, 2.93, and 2.94 log CFU/mL for SB, MB, and WB, respectively. A statistically significant (*p* < 0.05) decrease in the number of bacterial cells was evident after 10 min of inhibition by applying an electric current of 10 mA on the zirconium discs (time 0 min to 10 min) for SB (*p* = 0.013), MB (*p* = 0.0338), and WB (*p* = 0.002). After another 50 min (10 min to 60 min) of exposure to the current, no statistically significant results were found. However, the dependence of the biofilm reduction for the time 0 min to 60 min was statistically significant for each of the biofilms on the zirconium discs: SB, *p* = 0.0214; MB, *p* = 0.0003; and WB, *p* = 0.009. For the titanium discs in the inhibition of biofilm by electric current of 10 mA, the results for the time after 10 min (time 0 min to 10 min) were as follows: SB, *p* = 0.00009; MB, *p* = 0.006; and WB, *p* = 0.003. However, after another 50 min (10–60 min), only for SB (*p* = 0.0475) and MB (*p* = 0.037), the results were statistically significant. The reduction of biofilm formation from 0 min to 60 min was statistically significant in SB (*p* = 0.006), MB (*p* = 0.003), and WB (*p* = 0.001).

### 3.2. Effect of Low Amperage Electric Current of 1 mA in Eradication and Inhibition Assays on Biofilm Formation on Ti-6Al-4V and Zirconium Dioxide Discs

**Figure 3:**
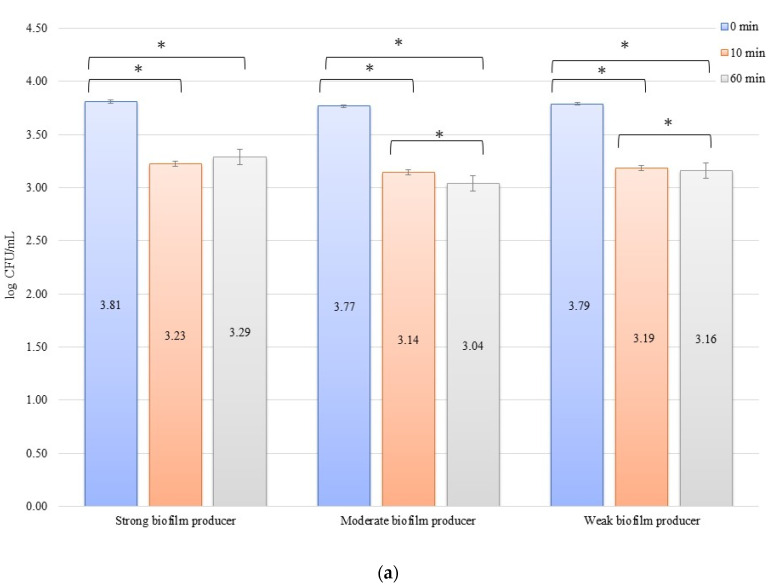

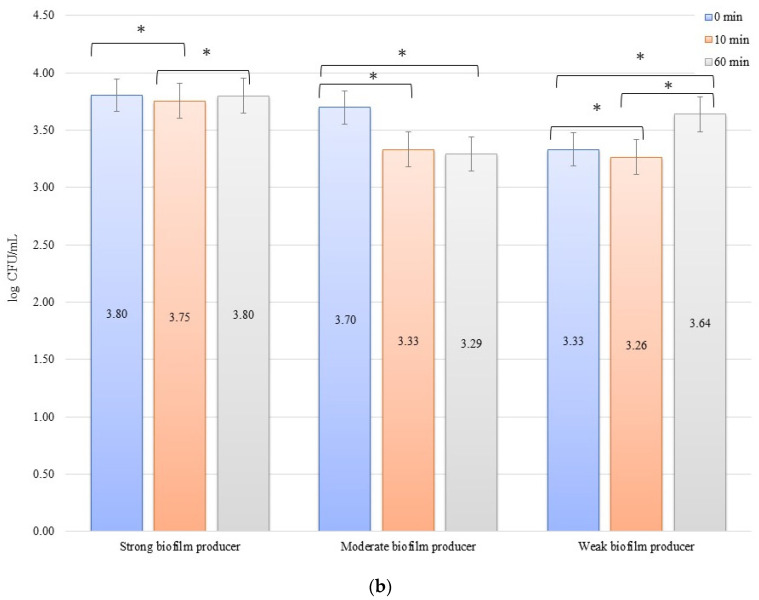
Biofilm eradication assay for *Staphylococcus aureus*. Strains forming biofilms on Ti-6Al-4V (**a**) and zirconium dioxide (**b**) discs classified as strong, moderate, weak, and non-producers are treated using low direct electric currents of 1 mA. Error bars represent the pooled standard deviations of the mean. The level of significance is presented at * *p* < 0.05. The mean and standard deviation are shown.

In an evaluation of the low current of 1 mA effect on biofilm formation on biomaterials, no significant decrease in the number of bacteria was observed over time after exposure to the current (Figure 3a,b). The initial numbers of bacteria in the experiments on biofilm erosion on the zirconium discs were 3.80, 3.70, and 3.33 log CFU/mL for SB, MB, and WB, respectively. For the titanium discs, these were 3.81, 3.77, and 3.79 log CFU/mL for SB, MB, and WB, respectively. The decrease in the number of cells was statistically significant (*p* < 0.05) after 10 min of erosion for each biofilm on the zirconium discs: SB, *p* = 0.018; MB, *p* = 0.0002; and WB, *p* = 0.0455. After 50 consecutive min (10 to 60 min), statistically significant results were obtained for SB (*p* = 0.018) and WB (*p* = 0.0007). However, for the time from 0 to 60 min, statistically significant results were obtained for MB (*p* = 0.0002) and WB (*p* = 0.005) only. For the titanium discs, the decrease in the number of bacterial cells in biofilms was statistically significant for the following times: after 10 min of erosion (SB, *p* = 0.003; MB, *p* = 0.0001; WB, *p* = 0.006), after 50 consecutive min (10 to 60 min) (MB, *p* = 0.002; WB, *p* = 0.038), and for 0 to 60 min (SB, *p* = 0.00001; MB, *p* = 0.0001; and WB, *p* = 0.001).

**Figure 4:**
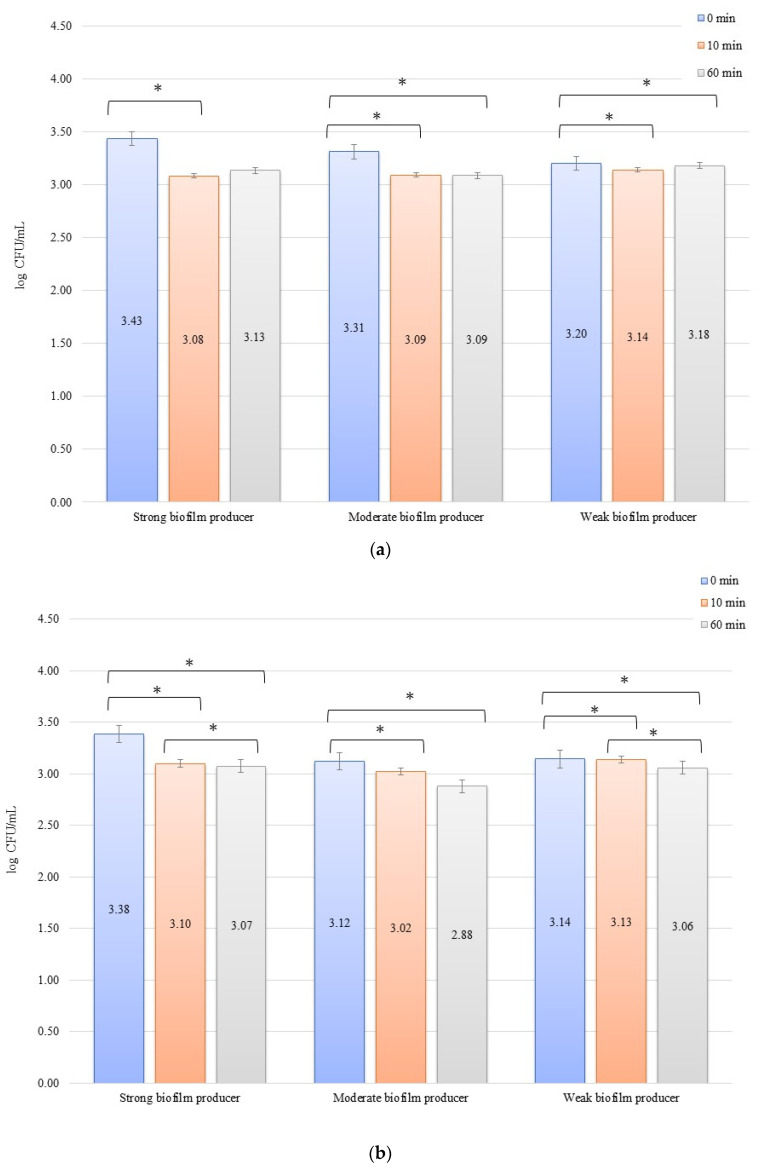
Biofilm inhibition assay for *Staphylococcus aureus*. Strains forming biofilms on Ti-6Al-4V (**a**) and zirconium dioxide (**b**) discs classified as strong, moderate, weak, and non-producers are treated with low direct electric currents of 1 mA. Error bars represent the pooled standard deviations of the mean. The level of significance is presented at * *p* < 0.05. The mean and standard deviation are shown.

In the inhibition studies, the initial numbers of biofilm cells (Figure 4b) on the zirconium discs showed the following increase in bacterial biofilm: 3.38, 3.12, and 3.14 log CFU/mL for SB, MB, and WB, respectively. On the titanium discs, these were as follows: 3.43, 3.31, and 3.20 log CFU/mL for SB, MB, and WB, respectively (Figure 4b). The results of the influence of low current on biofilm formation in the inhibition studies over time on the zirconium discs were statistically significant after 10 min of inhibition (0 to 10 min) for BD (*p* = 0.004) and MB (*p* = 0.01). After 50 min (10 to 60 min) for BD (*p* = 0.026) and WB (*p* = 0.038) and for 0 to 60 min, statistically significant results were obtained for SB (*p* = 0.005), MB (*p* = 0.01), and WB (*p* = 0.036). The decrease in the number of bacterial cells under the influence of current with a low intensity of 1 mA was statistically significant (*p* < 0.05) after 10 min of inhibition on the titanium discs (0 min to 10 min) for BD (*p* = 0.035), MB (*p* = 0.069), and WB (*p* = 0.040). After the next 50 min (10 to 60 min), no statistically significant differences were recorded, whereas for the time of 0 to 60 min, statistically significant results were obtained for MB (*p* = 0.008) and WB (*p* = 0.0004).

### 3.3. Effect of Low Amperage Electric Current of 1 mA and 10mA in Assays on Biofilm Formation in Fluorescence Microscopy

**Figure 5:**
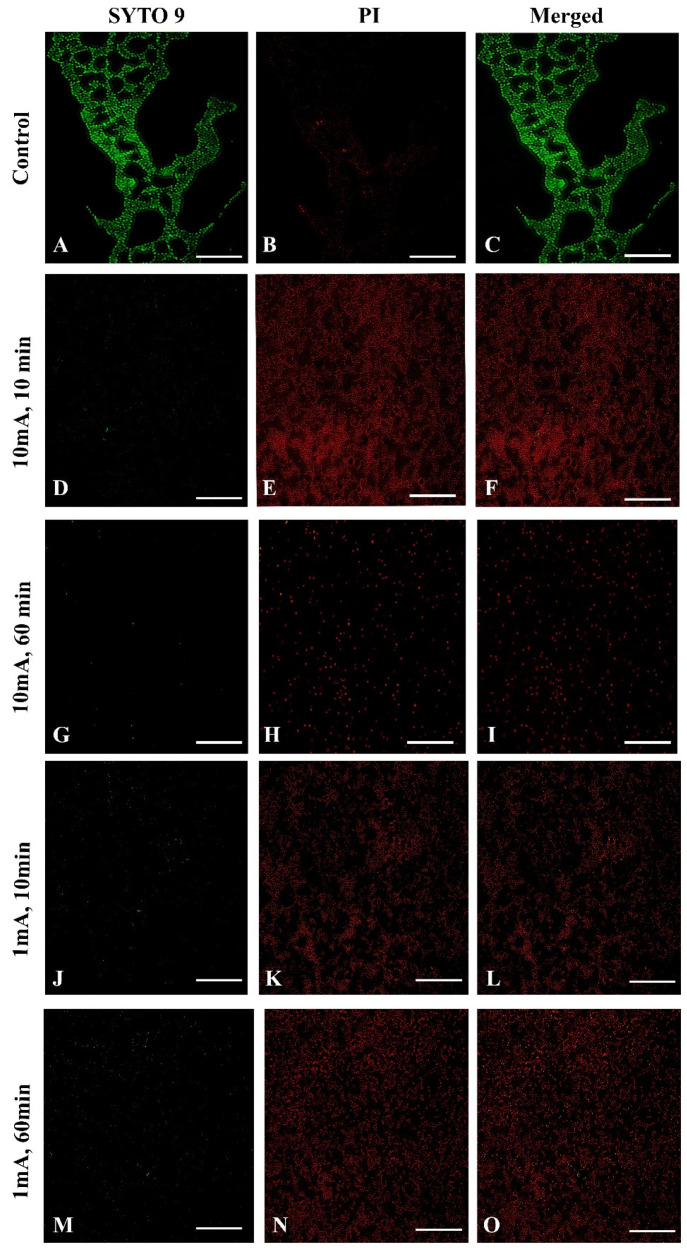
Fluorescence microscopy images of Live/Dead of *S. aureus* of biofilm eradication assay with low amperage electric current electrical stimulation, the green fluorescence (SYTO 9) indicates the live bacteria and red fluorescence (potassium iodide, PI) indicates the dead bacteria. (Scale bar = 50 μm). Control samples: (**A**) SYTO 9, live bacteria, (**B**) PI, dead bacteria, (**C**) a merged image of both types of staining; 10 mA after 10 min. (**D**) SYTO 9, (**E**) PI, (**F**) merged images; 10 mA after 60 min. (**G**) SYTO 9, (**H**) PI, (**I**) merged images; 1 mA after 10 min. (**J**) SYTO 9, (**K**) PI, (**L**) merged images; 1 mA after 60 min. (**M**) SYTO 9, (**N**) PI, (**O**) merged images.

To confirm the influence of low amperage electric current electrical stimulation on biofilm S. aureus we examined the biofilms with live/dead staining using fluorescence microscopy. To visualize influence of low amperage electric current electrical stimulation on biofilm S. aureus we used sterile glass coupons. Figure 5 shows a control (A–C) with strong green fluorescence showing live S. aureus cells. The LIVE/DEAD (SYTO 9/PI) staining visually confirmed a reduced live bacteria count and indicated significantly higher percentages of dead bacteria stained red (Figure 5E,H,K,N) after low amperage electric current electrical stimulation compared, to the control (Figure 5B). These results are consistent with the biofilm cell count results shown above. Nonetheless, after 60 min at 10 mA (Figure 5G–I) and 1 mA (Figure 5M–O), the damaged structure of the biofilm is visible, which is not visible after 10 min 10 mA (Figure 5D–F) and 1 mA (Figure 5J–L).

## 4. Discussion

This in vitro study analyzed the influence of low-intensity current on biofilm formation on Ti-6Al-4V and zirconium oxide biomaterials. Although these biomaterials were selected by us, they are also available and popular in dentistry. Our [34] laboratory studies have also shown that biofilms from *S. aureus* isolated from adult patients with CF can be reproducibly grown on the surface of implant biomaterials.

In recent years, dental implants have become a reliable solution for the rehabilitation of the oral cavity, allowing the replacement of missing teeth and tissues with reconstructions anchored on osseointegrated implants Nevertheless, since the introduction of this method in dentistry, there have been reports on complications that shortened the survival time of implants. Reported factors affecting implant survival time include changes in the oral microbiota, formation of biofilm, reaction of the host’s immune system to biomaterials, systemic diseases, periodontal diseases, peri-implant diseases, and the implant surface, which is not smooth resulting in increased osteointegration [37,38].

Observational studies have shown that peri-implantitis was more commonly associated with opportunistic pathogens, such as *P. aeruginosa* and *S. aureus*; fungi, such as *Candida albicans*, *Candida boidinii*, and *Penicillium* spp., and viruses, thereby, indicating a complex and heterogeneous infection [39,40]. Numerous scientific reports have shown that *S. aureus* was a frequent cause of chronic and resistant infections, especially in patients with CF and dental implants [41,42,43,44]. Moreover, infections associated with implant rejection were associated with biofilm formation by *S. aureus* on the surfaces of the hydrophilic implant than on hydrophobic materials, such as zirconium and titanium [45,46].

Our study revealed a major reduction in the formation of bacterial biofilms on the surface of the zirconium discs in comparison with titanium samples. Our observations are supported by other references and in vitro tests of bacterial adhesion to various surfaces of titanium and zirconium, where significantly fewer bacteria were observed on zirconium surfaces than on titanium surfaces [47,48]. In addition, Scaraono et al. [49] found significantly fewer bacteria on the zirconium than on titanium surfaces during the initial phase of adhesion. Several studies have emphasized that zirconium oxide and its derivatives (ZrN) not only have the ability to reduce plaque on the implant and surrounding tissues, which may play a major role in soft tissue healing and implant success but, above all, they allow to avoid bone resorption around the implant [50]. Conversely, Lima et al. [51] and Al-Ahmad et al. [52] reported that titanium and zirconium dioxide surfaces displayed similar biological properties in terms of protein adsorption, biofilm composition, and bacterial adhesion [51,52].

One particular method for controlling biofilms and disrupting biofilm formation, which has been widely discussed in the literature, is the use of physical stimulations (that is, applying low-intensity electric current on the biomaterial surface where the biofilm grows). This method seems noteworthy, particularly in the light of increasing reports in the literature that bacteria in mature biofilms can develop resistance to biocides and antibiotics [53,54], which requires 50–5000 times higher concentrations of antibiotics to achieve an effective extent of killing than that required for planktonic bacteria [55]. Therefore, the use of either constant electric potential or constant electric current that manipulates electrochemical phenomena on a target surface to combat biofilm and control the initial adhesion of bacteria to dental biomaterials can potentially represent a modifiable risk factor for avoiding infection and acquiring resistance to strains in patients with implants who have chronic infections, such as CF.

The use of low-intensity electrical current to control biofilm formation allows the control of the properties or reactions at the surface to delay and/or prevent cell adhesion or remove the existing cells from that surface. Since almost all biomaterials are negatively charged, as bacteria are, the electrostatic forces between bacteria and biomaterial surfaces are generally repulsive. The application of electric current may increase these repulsive forces, leading to the detachment of bacteria and bacterial biofilms from the surface of biomaterials [55,56,57]. Two in vitro experiments demonstrated that *S. aureus*, *Staphylococcus epidermidis*, and *P. aeruginosa* infections could be effectively treated using low-level direct current therapy [57,58]. In our study with *S*. *aureus* forming WBs, MBs, and SBs on the titanium and zirconium discs, we confirmed and noted similar observations of detachment and quantitative reduction of bacteria in the biofilm erosion and inhibition experiments on both biomaterials using 10 mA current after 10 min of application. The results with 1 mA also demonstrated statistically significant changes and reduction in the number of bacteria in the biofilms on both biomaterials. Nevertheless, the reduction was not as strong as that observed with 10 mA. We also observed that after 60 min of applying 1 mA, the number of bacteria remained unchanged, and even a slight increase in the number of bacteria was observed at this low intensity of current. This was not the case at 10 mA, when the number of bacteria in the biofilm was still reduced over time. Therefore, acute conditions/infections require prophylaxis to use a higher intensity to eliminate, rather than merely inhibit, bacterial growth.

Poortinga et al. [59] and Van der Borden et al. [60] also demonstrated that electricity stimulated the detachment of bacteria from biomaterials. Based on the knowledge that bacteria interact with surfaces by attracting Lifshitz-Van der Waals forces, acid-base interactions, and electrostatic forces, as shown in the Derjaguin, Landau, Verwey, and Overbeek theory and conductive (or semiconductive) materials, free electrons cause short-range electron exchange interactions, also known as metallic bonds [61]. The authors demonstrated that it was possible to stimulate the detachment of *Streptococcus oralis*, *S. epidermidis*, and *S. aureus.* The bacterial detachment mechanism presented by these research groups was based on the ionic strength dependence of electron transfer during the initial bacterial adhesion, which must be reversed to allow detachment. At the same time, the applied potential affects the peeling process since the peeling force is proportional to the tension and must overcome the attractive forces of Lifshitz-Van der Waals and acid-base forces between the adhering bacteria and the surface. Therefore, it was noted that detachment did not occur in the absence of any current, and only the use of electricity caused the detachment of bacteria from the surface, which was also confirmed in our study. After 10 min, the experiment with the 10 mA current in erosion for large, medium, and small biofilms revealed a reduction in the number of bacteria on the titanium and zirconium discs (Figure 1a,b). This proves the simultaneous inhibition of the adhesion of new bacteria to the surface of biomaterials and the commencement of detachment of the bacteria that were already present in the biofilm on the biomaterial surface (Figure 5). The same observation was made in our experiments for all strains in the inhibition assay using a current of 10 mA for both biomaterials (Figure 2a,b). The reduction of bacteria in these experiments was sustained over time, as evidenced by the 60 min of current exposure and a marked reduction in the number of bacteria on both the titanium and zirconium discs. Experiments with the use of 1 mA showed that the effect of inhibiting the adhesion and detachment of bacterial strains from biomaterials was rather ordinary, but it was not statistically significant. This may prove that the current used in the test was significantly low and the force was insufficient to detach the bacteria from the surface of the biomaterials. However, this process inhibited the adhesion of new bacteria to the surface from the environment, which could be observed after 10 min of exposure to the current (Figure 3a,b or Figure 4a,b). In both cases of erosion and inhibition, the number of bacteria in biofilms on the titanium and zirconium discs was slightly reduced after 10 min. However, after 60 min of the exposure to 1 mA, the number of bacteria was reduced only in the inhibition experiment with WB producer on the zirconium discs (Figure 4b), while in the other tested samples at an intensity of 1 mA, after 60 min, the number of bacteria was increased slightly. This increase confirms our assumption that 1 mA is significantly low to be used to combat biofilms on dental implants.

## 5. Conclusions

Oral health has not traditionally been an interest of the multidisciplinary cystic fibrosis team. Despite recent advances in the treatment of CF, challenges remain to improve dental management and new interventions are needed to improve the outcomes of the growing population of adult patients with CF. Therefore, our research, although not sup-ported by the results of clinical trials, could have a significant impact on the development of guidelines for the dental management of adult CF patient. The non-invasive method using low intensity direct current, the antimicrobial effectiveness of which we have demonstrated in our work, can be used both in prophylaxis and dental treatment of CF patients. In addition, the biomaterials used in dental implants, such as titanium and zir-conium can also significantly support this method of prophylaxis and treatment not only for this selected group of patients.

## Data Availability

Not applicable.
